# Univariate and Multivariate Pattern Analysis Reveals the Effects of Negative Body Image at Fatness on Food-Related Inhibitory Control

**DOI:** 10.3390/nu17152555

**Published:** 2025-08-05

**Authors:** Zihan Xu, Yuchan Xu, Junyao Han, Lechang Sun, Junwei Lian, Zhifang Li, Yong Liu, Jia Zhao

**Affiliations:** 1 Faculty of Psychology, Southwest University, Chongqing 400715, China; cz1248hx266@163.com (Z.X.); yuchan1936@outlook.com (Y.X.); psychologist@email.swu.edu.cn (J.H.); 15207210002@163.com (L.S.); ljw981846236@email.swu.edu.cn (J.L.); liuy0768@swu.edu.cn (Y.L.); 2 School of Psychology and Cognitive Science, East China Normal University, Shanghai 200062, China; lizhifang7@outlook.com; 3 Key Laboratory of Cognition and Personality (Ministry of Education), Southwest University, Chongqing 400715, China

**Keywords:** negative body image, inhibitory control, obesity, multivariate pattern analysis

## Abstract

Background/Objectives: Perceptions of obesity critically influence people’s eating behaviors and responses to food stimuli. However, few studies have investigated the impact of negative body perception on behavioral and neural responses to food stimuli. This study investigates how elevated body dissatisfaction modulates food-related inhibitory control. Methods: Fifty-one participants comprising three cohorts—overweight/obese individuals (OO), normal-weight participants exhibiting high negative body image (HNN), and healthy controls—performed a food-specific inhibitory control task under EEG recording. Results: The results showed that the HNN cohort achieved superior no-go accuracy and enhanced inhibitory control compared to controls. An event-related potentials (ERPs) analysis revealed increased conflict detection (P200) for high-calorie foods and reduced conflict resolution (LPP) in the HNN group, similar to the overweight/obese group. A multivariate pattern analysis (MVPA) identified earlier neural discrimination in the HNN group, suggesting more efficient inhibitory processing. Conclusions: These findings underscore negative body perception as a critical modulator of food-related cognitive control mechanisms.

## 1. Introduction

Obesity, pathologically characterized by excessive adipose accumulation detrimental to health outcomes [1,2], has emerged as a pressing global public health concern. While its association with metabolic disorders is well-established, mounting evidence implicates obesity in neurocognitive alterations, including executive dysfunction and structural deficits in prefrontal regulatory regions, e.g., the dorsolateral prefrontal cortex and the anterior cingulate cortex [3,4,5,6]. Central among these executive impairments is inhibitory control—the capacity to suppress prepotent responses in favor of goal-directed behavior [7]. Deficits in this domain often manifest as impulsive, stimulus-driven behaviors [7]. The go/no-go (GNG) paradigm has been widely validated as a neurobehavioral measure of inhibitory control [8]. Prior findings indicate that obese adolescents exhibit attenuated conflict monitoring during no-go trials and diminished attentional engagement during go trials relative to healthy-weight peers [9]. Critically, impaired inhibitory control has been identified as a prospective risk factor for the development of obesity [10]. For instance, Cui et al. demonstrated that reduced suppression of food-related thoughts, as assessed by the think/no-think task, was positively associated with visceral adiposity [11].

While these findings underscore a bidirectional relationship between obesity and inhibitory dysfunction, most prior work has focused exclusively on individuals with elevated body mass index (BMI), thereby overlooking the cognitive and neural consequences of psychological adiposity—specifically, negative body image among individuals with normative weight status. In contemporary sociocultural contexts that idealize thinness [12,13], body dissatisfaction and restrictive eating behaviors are increasingly prevalent, even among individuals within the healthy BMI range [14,15]. This subset, referred to here as individuals with high negative body image and normal weight (HNN), shares motivational and behavioral similarities with restrained eaters (REs), including persistent concerns about body shape and efforts to control weight despite normative body size.

Previous research has demonstrated that REs show altered cognitive processing in response to food cues, including enhanced inhibitory control during food-specific GNG tasks [16] and attentional vigilance followed by rapid disengagement from food stimuli [17]. These findings raise an important question: do HNN individuals similarly exhibit distinctive inhibitory control profiles compared to body-satisfied normal-weight individuals (i.e., those with low NPSS-F scores)? Notably, many existing studies classify HNN participants as healthy controls based solely on BMI, potentially obscuring meaningful neurocognitive heterogeneity within the normal-weight population. Addressing this limitation, the present study aims to clearly define an HNN group and employ a neurocognitive approach with both behavior and electroencephalography (EEG) measures to investigate food-specific inhibitory control across three distinct groups: HNN, overweight/obese (OO), and body-satisfied control groups (CON).

Electroencephalography (EEG), particularly event-related potentials (ERPs), provides millisecond resolution indices of neural dynamics during cognitive processing [18]. In dietary and obesity research, several ERP components have been identified as informative neural markers. The P200 component reflects early attentional vigilance, with amplitude potentiation linked to negative affective traits during salient/threatening stimulus processing [19,20]. N200 is a negative fluctuation around 200 ms after the appearance of stimuli, which is a putative marker of conflict monitoring and response inhibition, though its functional interpretation remains debated. Amplitude enhancements are reported during inhibition/conflict [21,22], while some paradigms show larger amplitudes in go trials [23]. P300 is a positive-going ERP component peaking around 300 ms post-stimulus, which can be subdivided into P300a and P300b subcomponents in specific paradigms, indicating attentional reorientation and context updating/resource allocation during inhibitory control and working memory, respectively [15]. Late positive potential (LPP) reflects sustained motivational salience processing, including appetite/aversive engagement [24].

Despite the utility of ERPs, many existing studies on obesity-related inhibitory control focus narrowly on single components (e.g., N200, P300) or limited electrode sites (e.g., Fz), potentially overlooking broader spatiotemporal patterns of brain dynamics. Multivariate pattern analysis (MVPA) addresses this limitation by decoding distributed neural activation patterns across the entire scalp, offering enhanced sensitivity to subtle cognitive state differences [25,26,27]. As such, MVPA provides a complementary systems-level perspective on neural architecture supporting inhibitory control.

This study integrates ERP and MVPA approaches to comprehensively investigate food-specific inhibitory control across the OO, HNN, and CON groups. We hypothesize the following: (1) OO individuals will exhibit reduced accuracy and prolonged reaction times on complex food–GNG trials relative to CON, whereas HNN individuals will demonstrate enhanced accuracy and expedited responses; (2) OO individuals will display attentional bias (elevated P200 amplitude) and heightened resource allocation (elevated P300 amplitude) toward food stimuli, while HNN individuals will show increased attentional vigilance (elevated P200) and enhanced conflict resolution/control (modulated N200/P300 complex) attributable to chronic body dissatisfaction; (3) both the OO and HNN groups will exhibit distinct, temporally extended neural activation patterns during go/no-go conditions compared to CON, detectable via whole-scalp MVPA across the EEG time series.

## 2. Materials and Methods

### 2.1. Participants

A total of 51 undergraduate students from Southwest University were recruited for this study. All participants were aged between 18 and 22 years, reported good health, had normal or corrected vision, and had no history of neurological or psychological disorders. Based on the measured BMI and NPSS-F scores, the participants were categorized into three groups: the overweight/obese group (9 males in 18 participants, BMI ≥ 24 kg/m^2^; 12 participants qualified as overweight, with BMI scores in [24, 28] kg/m^2^, and 6 participants qualified as obese, with BMI scores ≥ 28 kg/m^2^, according to China’s obesity standard [28]); the HNN group (7 males in 17 participants, BMI scores in [18.5, 23.9] kg/m^2^, NPSS-F > 2.5); and the control group (9 males in 16 participants, BMI scores in [18.5, 23.9] kg/m^2^, NPSS-F < 1.5). Notably, the HNN group exhibited elevated fat-related self-perception despite having BMI values comparable to those of the control group, making it suitable for investigating the impact of body image on food-related cognitive control. Importantly, BMI was measured directly, offering greater accuracy than self-reported values. All participants gave written informed consent before the beginning of the experiment. Ethical approval for the study was granted by the Institutional Review Board of Southwest University (approval number: H23047).

### 2.2. Materials

#### 2.2.1. Self-Measurements

The participants were required to evaluate their hunger level, thirst level, and desire to eat using a visual analog scale (VAS) [15]. The questions were as follows: “How hungry are you now?” “How thirsty are you now?” and “How strong is your desire to eat now?”. The responses ranged from “not at all” to “very strong”, with the average of the three ratings utilized as the index of the participant’s hunger state.

The restrained eating subscale of the Dutch Eating Behavior Questionnaire (DEBQ) was used to assess the participants’ eating behaviors [29]. It includes 10 items, such as “Do you eat less than usual when you gain weight?”. The participants’ responses ranged from “never (1)” to “always (5)”, with larger scores suggesting stronger dietary restraint and greater eating self-control. This subscale has demonstrated strong internal consistency across different weight groups, with Cronbach’s α ranging from 0.92 to 0.94 in non-clinical samples [30].

The Negative Physical Self Scale (NPSS), which measures body satisfaction across emotional, cognitive, and behavioral dimensions, was used to assess dissatisfaction with body image [31]. The fatness subscale contains 11 items and uses a 5-point Likert scale, with increasing scores from 0 to 4 indicating “never” to “always”. A higher score on the NPSS-F indicates stronger dissatisfaction with one’s body weight or a perception of being overweight. Participants with an average score of more than 2.5 were classified as having a negative body image, consistent with grouping criteria from prior research [32].

The Positive and Negative Affect Schedule (PANAS) scale was used to assess the current emotional state of the participants [33]. The scale includes 20 items that are based on a 5-point Likert scale, ranging from “not at all (1)” to “extremely (5)”. The scale is divided into two dimensions: positive affect and negative affect, with the total scores calculated for each dimension separately.

The food pictures in the go/no-go task were selected from the Chinese food image database [34], which was rated and standardized by individuals from senior high schools and colleges. It was divided into high- and low-calorie foods according to the food’s caloric value.

#### 2.2.2. Experimental Procedure

The study employed a 3 (group: OO, HNN, CON) × 2 (food stimulus: high-calorie, low-calorie) × 2 (task type: go, no-go) mixed experimental design.

**Figure 1:**
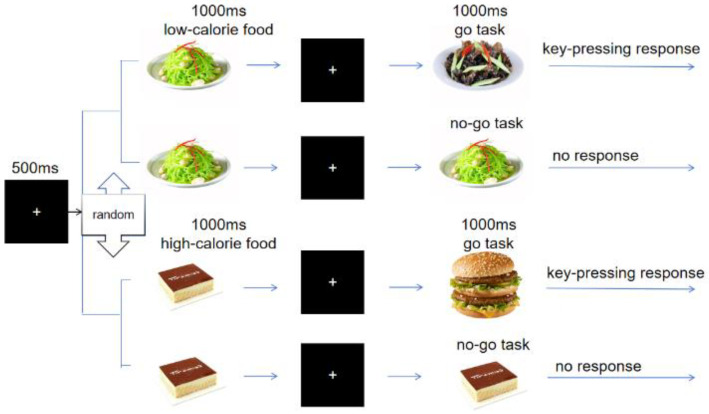
Flowchart of food-related go/no-go task.

The experimental task was a food-related go/no-go task. In this task, the participants were asked to make a key-press response only when the food stimulus was the same as the previous one. If the neighboring stimulus was different, no response was required. The task procedure is illustrated in Figure 1. The experiment was divided into two blocks (120 trials for each block), with a go/no-go ratio of 2:1 (160 go trials, including 80 high-/low-calorie food cues, respectively, and 80 no-go trials, including equal food cues at high- or low-calorie). A one-minute resting period separated the two parts of the experiment. The participants were instructed to keep their heads fixed and minimize blinking during the task. Each trial began with a fixation point displayed for 500 ms, followed by a randomly presented food stimulus (either high-calorie or low-calorie) for 1000 ms. If a participant pressed the response key, the food stimulus disappeared, and a blank screen appeared. The task duration was approximately 10 min, during which EEG data were continuously recorded. The participants were compensated with a 60 RMB fee for their participation.

Prior to the experiment, the participants completed the NPSS-F questionnaire online and provided their gender and BMI to judge whether they met the inclusion criteria. After obtaining informed consent, the participants completed the hunger, thirst, desire to eat, DEBQ-RS, and PANAS questionnaires. Next, they were required to complete the food-related go/no-go task.

#### 2.2.3. Data Recording and Analysis

(1) Behavior Analyses

The mean (M) and standard deviation (SD) of the self-reported variables for each group were calculated. To assess group differences on the demographic and questionnaire measures, one-way analysis of variance (ANOVA) was calculated for these variables.

For the response accuracy (ACC_Go and ACC_No-go), a three-way repeated-measures ANOVA was employed using the following factors: 3 (subject group: overweight/obese, HNN, and control) × 2 (food type: high-/low-calorie) × 2 (task type: go, no-go). Here, the three groups were a between-subjects factor, while the food type and task type were within-subject factors. All statistical analyses were performed using IBM SPSS 27 (IBM, Armonk, NY, USA).

(2) EEG Recording and Analyses

The EEG data were captured using the NeuSen W series wireless EEG system with a 1000 Hz sampling rate and 64 Ag/AgCl electrodes placed on the scalp (Neuracle Technology Co., Ltd., Changzhou, China). The EEG data analysis was performed with EEGLAB v2022.0 (Swartz Center for Computational Neuroscience, La Jolla, CA, USA) using MATLAB R2022a software (The MathWorks, Inc., Natick, MA, USA).

The EEG data were initially filtered with a 1–30 Hz bandpass finite impulse response (FIR) filter. Subsequently, EEG epochs were extracted from −200 to 1000 ms relative to the stimulus onset. Each trial was visually inspected, and those with large amplitudes were excluded. Independent component analysis (ICA) was then applied to the remaining data to remove artifacts from eye blinks, head movements, electrocardiogram, and channel noise [35,36]. Following artifact removal, the trials were re-examined to ensure that no remaining poor-quality trials existed [14,36]. The averaged ERP waveforms of three electrodes (F3, Fz, and F4) at the frontal cortex for each group (overweight/obese, NPSS-F, and control) were calculated, during which the baseline reference was selected between −200 ms and 0 ms.

Statistical analysis of the ERP data was performed using SPSS 27, with a three-factor repeated-measures ANOVA (RMANOVA) conducted on the average amplitudes of the P200 (150–200 ms), N200 (210–260 ms), P300 (270–330 ms), and LPP (440–540 ms) components. The design for the RMANOVA was the same as for the behavioral part. Post hoc tests or simple-effects analyses were performed where necessary based on the results of the RMANOVA.

Multivariate pattern analysis (MVPA) was performed based on the Amsterdam Decoding and Modeling Toolbox [37]. The goal of this analysis was to determine whether neural activity patterns in the brain could distinguish between go and no-go stimuli following their presentation. The analysis period ranged from 200 ms prior to the stimulus onset to 1000 ms post-stimulus. The EEG activities measured by electrodes across the entire scalp were considered for analysis. The go and no-go conditions served as the classification categories in the MVPA.

A five-fold cross-validation procedure was employed to train and evaluate the classification of each participant’s electrophysiological responses recorded via EEG, utilizing Linear Discriminant Analysis (LDA). The dataset was randomly partitioned into five subsets with an equal distribution of trials. In each cross-validation iteration, 80% of the trials were designated as the training set, while the remaining 20% served as the testing set. This procedure was repeated five times, ensuring that each subset functioned as the testing set exactly once. The classification performance was quantified by calculating the area under the curve (AUC), which reflects the efficacy of the neural signal discrimination over time for each response category and participant. The AUC values ranged from 0 to 1, with a chance level of 0.5. To assess the statistical significance of the observed classification performance at the group level, a cluster-based permutation test was conducted comparing the average AUC against the chance level. Moreover, the mean AUC within the 0–1000 ms post-stimulus window—during which the go and no-go trials were successfully decoded—was extracted for each participant. A one-way repeated-measures ANOVA was then performed to compare the classification accuracy across the three groups. Additionally, a temporal generalization analysis was carried out by training the classifier at each time point and testing it across all other time points, in order to evaluate the temporal stability of neural activity patterns. Classification accuracy values exceeding chance levels off the diagonal line indicated the presence of stable neural representations over time.

## 3. Results

### 3.1. Self-Reported Results

One-way analysis of variance (ANOVA) was carried out for each variable. The results in Table 1 indicate significant differences in scores on the DEBQ-RS, the NPSS-F, and the BMI. However, no significant differences were found for the other variables.

Post-hoc analyses using the Least Significant Difference (LSD) correction showed that the BMI of the overweight/obese group was much higher in contrast to the control group (*p* < 0.001).

### 3.2. Behavioral Performance

The mean accuracy (ACC) and response time (RT) for the three groups in the go/no-go task were calculated (Table 2). A three-way repeated-measures ANOVA on RT revealed a significant main effect for food type (F = 16.178, *p* < 0.001). It showed that low-calorie food elicited a longer RT compared with high-calorie food.

A three-way repeated-measures ANOVA on ACC found a significant main effect of food type (F = 11.432, *p* < 0.001), a significant main effect of task type (F = 125.252, *p* < 0.001), a significant interaction between task condition and group (F = 3.619, *p* = 0.034), and a significant interaction between group, food type, and task condition (F = 3.647, *p* = 0.034). It showed that the accuracy for food cues with high-calorie values was much higher when compared with food cues of low-calorie values (*p* < 0.001), and the ACC for the go task was significantly higher when compared to the no-go task (*p* < 0.001). The simple-effects analysis revealed that the ACC of the HNN group was much higher in contrast to the control group under the no-go condition (*p* = 0.025), and the ACC under the go condition was significantly higher than the no-go condition for each group (*p* < 0.001). Meanwhile, the simple-effects analysis for the three-factor interaction showed that the ACC of the control group was much higher than that of the HNN group (*p* = 0.043) under the go condition with low-calorie food stimuli, while the ACC of the control group under the no-go condition was lower than that of the HNN group for low-calorie food stimuli (*p* = 0.010).

### 3.3. ERP Results

**Figure 2:**
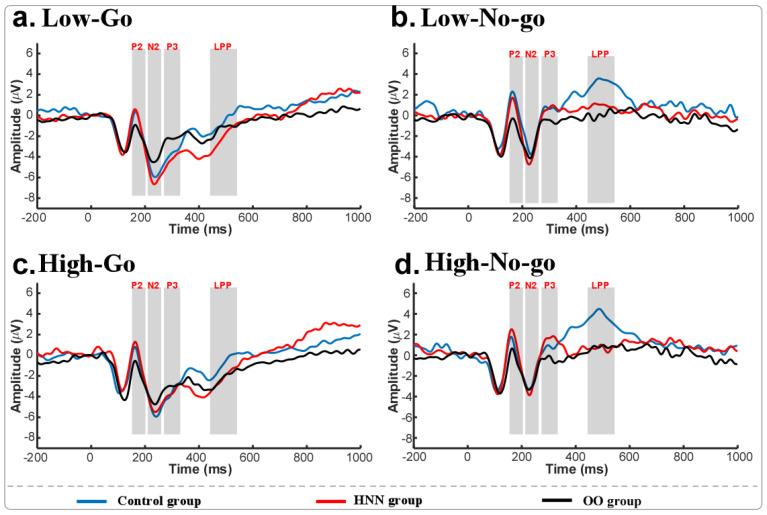
ERPs under different stimulus conditions for three groups.

The ERP results for the go and no-go conditions among the three groups following the presentation of food stimuli are shown in Figure 2. The three-factor repeated-measures ANOVA for the P2 amplitude found a significant main effect between the go and no-go conditions (F = 21.457, *p* < 0.001) and an interaction between low-/high-calorie food and groups (F = 3.276, *p* = 0.046).It showed that the P200 for the no-go trials was higher than for the go trials. Simple-effects analyses indicated that the P2 amplitude under the high-calorie food was higher than that for the low-calorie food in the HNN group (*p* = 0.018), but this effect was not found in the normal and obese groups.

The three-factor RMANOVA for the N2 amplitude found a significant main effect between high-/low-calorie food cues (F = 6.696, *p* = 0.013) and a main effect between the go and no-go trials (F = 40.391, *p* < 0.001). It showed that N2 under low-calorie food was more negative than under high-calorie food, and N2 under the go condition was more negative than under the no-go condition. The simple-effects analysis showed that N2 under the go trials was more negative than under the no-go trials in the CON (*p* < 0.001), HNN (*p* < 0.001), and OO (*p* = 0.027) groups.

The RMANOVA on the P3 amplitude revealed a main effect between the go and no-go trials (F = 84.162, *p* < 0.001) and an interaction effect between the three groups and the go/no-go trials (F = 4.384, *p* = 0.018). It showed that the P3 amplitude under the no-go condition was higher than under the go condition. The simple-effects test found that the P3 under the no-go condition was higher than under the go condition for the control (*p* < 0.001), HNN (*p* < 0.001), and obese (*p* = 0.004) groups.

The RMANOVA for the LPP component revealed a significant main effect between the go/no-go trials (F = 97.542, *p* < 0.001), a marginally significant main effect between groups (F = 2.943, *p* = 0.062), a significant interaction effect between low-/high-calorie food and go/no-go conditions (F = 4.099, *p* < 0.048), and a significant interaction effect between the go/no-go trials and the three groups (F = 3.685, *p* = 0.032). The post-hoc tests showed that LPP in the control group was higher compared to the HNN (*p* = 0.038) and obese (*p* = 0.041) groups, and LPP during the no-go trials was much higher than during the go trials. The simple-effects tests found that LPP for the no-go trials was higher compared to the go trials in each group (*p* < 0.001), LPP under the no-go condition in the control group was larger than in the HNN (*p* = 0.017) and obese (*p* = 0.006) groups, and LPP under the no-go condition was higher than under the go condition under both low- (*p* = 0.006) and high-calorie food (*p* < 0.001).

### 3.4. MVPA Results

**Figure 3:**
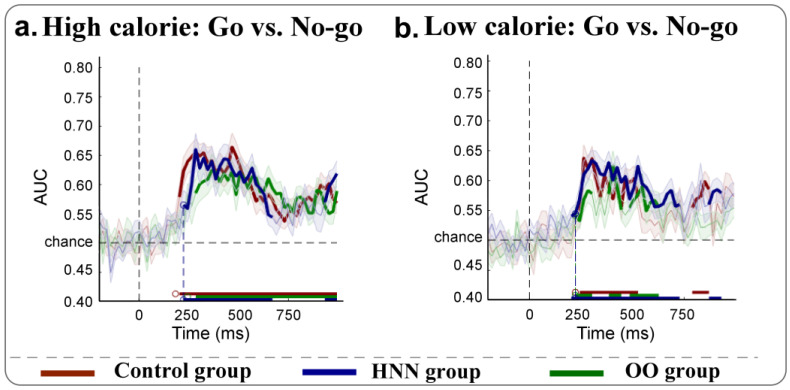
MVPA results in decoding neural dynamics between go and no-go trials for food cues with different calories.

The MVPA analysis revealed significant above-chance classification differences in decoding go and no-go conditions among the three groups following the presentation of food stimuli (Figure 3). For high-calorie food stimuli, the control group exhibited sustained successful decoding of go and no-go conditions across extended temporal intervals (220 to 1000 ms; *p* = 0.0049) compared to the other groups (obese group: 300 to 1000 ms, *p* = 0.0035; HNN group: 240 to 680 ms, *p* = 0.0042) due to the longer and more continuous decoding along the temporal axis. Conversely, under low-calorie food stimuli, the HNN group demonstrated prolonged successful decoding (220 to 740 ms and 880 to 940 ms, *p* = 0.0041) relative to the other groups (normal group: 260 to 540 ms and 800 to 880 ms, *p* = 0.0052; obese group: 240 to 320 ms, 400 to 460 ms, and 500 to 620 ms, *p* = 0.0122).

**Figure 4:**
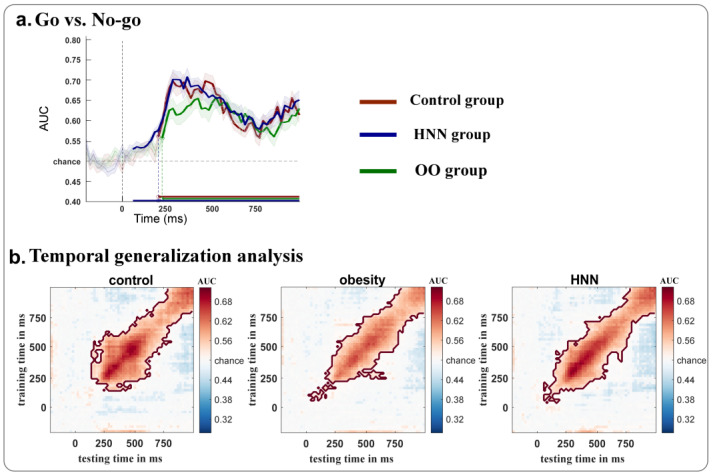
MVPA results in decoding neural dynamics between go and no-go trials without distinguishing food cues with different calories.

To further examine group differences in go/no-go classification irrespective of food type (Figure 4a), we observed that all groups showed more sustained decoding performance compared to their food-specific decoding, where significant AUC was observed in the normal group (240 to 1000 ms; *p* = 0.0021), obese group (240 to 1000 ms; *p* = 0.0029), and HNN group (180 to 1000 ms; *p* = 0.0026). Notably, the HNN group achieved pronounced above-chance classification at an earlier temporal range than the other groups. Temporal generalization matrices were computed to show the stability of the decoding results (Figure 4b). The control group exhibited the most stable classification performance, as indicated by the broader temporal extension of its generalization matrix beyond the diagonal axis. In contrast, both the HNN and OO groups showed earlier classification onset, with the OO group exhibiting the least stable classification, evidenced by reduced matrix intensity relative to the other groups.

## 4. Discussion

This study provides a comprehensive investigation of food-related inhibitory control and its underlying neural mechanisms across three groups: overweight/obese individuals, those with HNN, and healthy controls. Consistent with previous findings, the participants exhibited lower accuracy in no-go trials compared to go trials and responded more rapidly to high-calorie than low-calorie food stimuli. Notably, the HNN group demonstrated significantly higher no-go accuracy relative to the control group, suggesting enhanced inhibitory control over food-related cues. Moreover, this group showed higher accuracy in no-go trials involving low-calorie food stimuli compared to controls, while their performance in go trials for these same stimuli was reduced. These behavioral patterns were further supported by distinct electrophysiological signatures and MVPA, underscoring the unique cognitive and neural processing characteristics of individuals with high negative self-perception related to body image.

### 4.1. Distinct ERP Characteristics Among Groups in Response to Food Stimuli

The P200 and N200 ERP components are well-established indicators of early conflict detection and response inhibition [19,20,38], while the P300 and LPP components reflect the allocation of attentional and cognitive resources during conflict resolution [39]. Significantly enhanced P200 amplitudes were observed in the HNN group when processing high-calorie versus low-calorie food stimuli—an effect not observed in the control or overweight/obese groups—suggesting heightened neural sensitivity to food-related conflict detection.

Additionally, LPP amplitudes were significantly reduced in the HNN group compared to controls, mirroring patterns observed in the overweight/obese group. This reduction may reflect diminished regulatory capacity in response to food stimuli, possibly due to the sustained cognitive effort required by individuals with long-term dietary restrictions to reconcile their eating behaviors with body image concerns. These ERP findings align with the observed behavioral differences, providing a potential neural basis for the enhanced inhibitory performance seen in the HNN group.

Although no between-group differences were found for the P300 component, all groups demonstrated greater P300 responses during no-go versus go trials—consistent with the elevated cognitive demands associated with inhibitory control. Previous research identified an interaction effect on the P3a subcomponent, wherein the control group exhibited greater amplitudes for low-calorie stimuli compared to the HNN and overweight/obese groups [15]. However, such an interaction was not reflected in the overall P300 amplitude across go/no-go conditions in this study.

### 4.2. MVPA Decoding of Neural Dynamics During Food Stimulus Processing

MVPA has become a powerful tool for investigating cognitive control processes such as working memory [40] and inhibitory control [41], although its use in obesity-related inhibitory control remains limited. Our prior work demonstrated that theta-band activity could successfully distinguish go from no-go conditions, but not food type [27]. To improve temporal resolution and mitigate the influence of pre-defined time windows in time-frequency analyses, MVPA was applied to decode dynamic neural responses from 200 ms pre-stimulus to 1000 ms post-stimulus.

All groups demonstrated distinct neural patterns for go versus no-go trials, with decoding accuracy emerging around 250 ms post-stimulus onset in the control and overweight/obese groups. In contrast, the HNN group exhibited significantly earlier decodability, beginning as early as 80 ms post-stimulus. This rapid neural processing may enable earlier detection of food cues and facilitate superior inhibitory control during no-go trials, potentially accounting for the behavioral advantages observed. These findings are consistent with previous research showing improved inhibitory control among restrained eaters [16] and early attentional engagement with food cues in similar populations [42].

Attempts to decode specific stimulus categories (e.g., high- versus low-calorie) did not yield significant results in any group; therefore, these analyses are not discussed in detail.

### 4.3. Limitations and Future Directions

The functional interpretation of the N200 component remains uncertain due to inconsistencies in the literature. While some studies report enhanced N200 amplitudes for no-go versus go conditions [43,44], others show the opposite pattern [9,45]. In this study, larger N200 responses were observed for go trials and low-calorie stimuli, suggesting modulation by multiple factors.

Three key elements may explain these discrepancies. First, the go/no-go ratio is known to influence N200 amplitudes. For example, Donkers and Van Boxtel (2004) found that an 80% go-trial proportion enhances N2 amplitudes during no-go trials [21]. Our 2:1 go/no-go ratio may have contributed to enhanced N200 activity during go trials. Second, the task complexity design—which combined a go/no-go paradigm with a one-back task—likely increased cognitive demands and influenced ERP characteristics. Third, stimulus features may have played a role. The visual homogeneity of low-calorie foods (mostly green vegetables) compared to the more diverse high-calorie foods may have increased perceptual difficulty and influenced response time and ERP amplitudes.

These findings highlight the importance of considering the HNN population as a distinct group within obesity-related research, given their unique behavioral and neural profiles. Future studies should further explore N200 modulation in food-related tasks by incorporating the following: (1) systematically varying go/no-go ratios, (2) controlling for task complexity, and (3) accounting for visual characteristics of food stimuli.

Furthermore, the relatively small sample size in this study limits the generalizability of the findings. Larger and more diverse cohorts will be necessary to validate and extend these results. Future research should also consider recruiting individuals with elevated BMI but low NPSS-F scores to explore their cognitive responses to food cues. While such individuals may be more difficult to recruit—particularly in cultures where thinness is idealized—understanding their cognitive profiles could offer valuable insights into the dissociation between body image and behavioral regulation.

## 5. Conclusions

This study successfully validated several key hypotheses regarding the behavioral and neural characteristics of individuals with high negative body self-perception (HNN). First, significant group differences were found in behavioral performance: the HNN group showed higher accuracy in no-go trials than other groups, indicating enhanced inhibitory control in response to food-related stimuli. Second, the EEG analyses revealed that, compared to the control group, the HNN group showed increased P200 amplitude and decreased LPP amplitude. These neural differences may reflect alterations in conflict detection and resolution processes in the HNN group. Moreover, MVPA indicated earlier cognitive processing in the HNN group, suggesting heightened visual sensitivity to food stimuli—potentially linked to their daily habitual restrained eating behaviors.

Collectively, these findings highlight possible cognitive and neural alterations with high negative body self-perception. The results have important implications for future research aiming to understand and modulate food-related cognitive processes and body image concerns in this population. Further investigations using large samples and multimodal neuroimaging techniques are recommended to improve the robustness and depth of these findings.

## Figures and Tables

**Table 1:** Participant characteristics and self-reported measures.

Variables (M ± SD)	Control Group N = 16	HNN Group N = 17	OO Group N = 18	F	*p*
Age	19.38 (1.67)	19.82 (1.47)	19.78 (1.35)	0.448	0.64
DEBQ-RS ***	2.41 (0.81)	3.69 (0.68)	3.30 (0.82)	11.86	<0.001
NPSS-F ***	1.31 (0.21)	2.96 (0.33)	2.97 (0.38)	151.6	<0.001
Hunger	20.63 (16.52)	32.35 (15.62)	27.22 (17.76)	2.04	0.14
Thirst	41.25 (18.57)	36.47 (10.57)	32.22 (13.09)	1.68	0.198
Desire to eat	25.00 (18.26)	30.59 (17.13)	24.44 (19.17)	0.594	0.56
PAS	2.98 (0.73)	2.72 (0.58)	3.00 (0.61)	0.969	0.387
NAS	1.84 (0.72)	1.96 (0.70)	1.86 (0.63)	0.149	0.862
BMI ***	20.30 (1.38)	21.40 (1.44)	26.82 (1.88)	83.13	<0.001

Note: *** *p* < 0.001. Control Group: normal-weight individuals with low NPSS-F; HNN Group: normal-weight individuals with high NPSS-F; OO Group: overweight/obese group.

**Table 2:** Behavioral responses to food-related go/no-go task.

Variable	Control Group (M ± SD) N = 16	HNN Group (M ± SD) N = 17	OO Group (M ± SD) N = 18
RT_high	466.20 (55.81)	478.68 (72.43)	472.43 (56.48)
RT_low	474.13 (54.86)	486.12 (74.02)	479.90 (60.99)
ACC_low_nogo	0.877 (0.069)	0.929 (0.046)	0.910 (0.052)
ACC_low_go	0.989 (0.011)	0.977 (0.022)	0.983 (0.016)
ACC_high_nogo	0.915 (0.051)	0.936 (0.046)	0.922 (0.045)
ACC_high_go	0.991 (0.012)	0.989 (0.019)	0.994 (0.008)

## Data Availability

The data presented in this study are available on request from the corresponding author. The data are not publicly available due to concerns about privacy and ethics in personal decision-making.
